# Impaired endocytosis in proximal tubule from subchronic exposure to cadmium involves angiotensin II type 1 and cubilin receptors

**DOI:** 10.1186/1471-2369-14-211

**Published:** 2013-10-05

**Authors:** Mitzi Paola Santoyo-Sánchez, José Pedraza-Chaverri, Eduardo Molina-Jijón, Laura Arreola-Mendoza, Rafael Rodríguez-Muñoz, Olivier Christophe Barbier

**Affiliations:** 1 Departamento de Toxicología, Centro de Investigación y de Estudios Avanzados del Instituto Politécnico Nacional (CINVESTAV-IPN), Mexico City, México; 2 Departamento de Biología, Facultad de Química, Universidad Nacional Autónoma de México (UNAM), Mexico City, México; 3 Departamento de Fisiología, Biofísica y Neurociencias, Centro de Investigación y de Estudios Avanzados del Instituto Politécnico Nacional (CINVESTAV-IPN), Mexico City, México; 4 Departamento de Biociencias e Ingeniería, Centro Interdisciplinario de Investigaciones y Estudios sobre Medio Ambiente y Desarrollo del Instituto Politécnico Nacional (CIIEMAD-IPN), Mexico City, México

**Keywords:** Cadmium, Subchronic exposure, Endocytosis, Megalin, Cubilin, Angiotensin II type 1 receptor, Losartan

## Abstract

**Background:**

Chronic exposure to low cadmium (Cd) levels produces urinary excretion of low molecular weight proteins, which is considered the critical effect of Cd exposure. However, the mechanisms involved in Cd-induced proteinuria are not entirely clear. Therefore, the present study was designed to evaluate the possible role of megalin and cubilin (important endocytic receptors in proximal tubule cells) and angiotensin II type 1 (AT1) receptor on Cd-induced microalbuminuria.

**Methods:**

Four groups of female Wistar rats were studied. Control (CT) group, vehicle-treated rats; LOS group, rats treated with losartan (an AT1 antagonist) from weeks 5 to 8 (10 mg/kg/day by gavage); Cd group, rats subchronically exposed to Cd (3 mg/kg/day by gavage) during 8 weeks, and Cd + LOS group, rats treated with Cd for 8 weeks and LOS from weeks 5–8. Kidney Cd content, glomerular function (evaluated by creatinine clearance and plasma creatinine), kidney injury and tubular function (evaluated by Kim-1 expression, urinary excretion of N-acetyl-β-D-glucosaminidase (NAG) and glucose, and microalbuminuria), oxidative stress (measured by lipid peroxidation and NAD(P)H oxidase activity), mRNA levels of megalin, expressions of megalin and cubilin (by confocal microscopy) and AT1 receptor (by Western blot), were measured in the different experimental groups. Data were analyzed by one-way ANOVA or Kruskal-Wallis test using GraphPad Prism 5 software (Version 5.00). P < 0.05 was considered statistically significant.

**Results:**

Administration of Cd (Cd and Cd + LOS groups) increased renal Cd content. LOS-treatment decreased Cd-induced microalbuminuria without changes in: plasma creatinine, creatinine clearance, urinary NAG and glucose, oxidative stress, mRNA levels of megalin and cubilin, neither protein expression of megalin nor AT1 receptor, in the different experimental groups studied. However, Cd exposure did induce the expression of the tubular injury marker Kim-1 and decreased cubilin protein levels in proximal tubule cells whereas LOS-treatment restored cubilin levels and suppressed Kim-1 expression.

**Conclusion:**

LOS treatment decreased microalbuminuria induced by Cd apparently through a cubilin receptor-dependent mechanism but independent of megalin.

## Background

Cadmium (Cd) is a heavy metal found in the earth’s crust associated with lead, zinc and copper. Currently, this metal has become an environmental and public health problem due to its constant release by industrial activity. However, Cd is used for the manufacture of batteries, pigments, consumer electronics, and quantum dots [1,2]. This metal enters the food chain through contaminated air, water, and soils where it is caught and fixed by plants (ie. sunflower kernels and rice), molluscs and crustaceans (Cd contents >1-2 mg/kg ww) [3-5]. In consequence, general population is exposed to Cd by contaminated water and food [4,6]. Another important source of Cd is cigarette smoke at a concentrations of 1–2 μg Cd per cigarette. Smokers have more Cd in blood and kidneys than non-smokers. Smoking a pack of cigarettes per day leads to an absorption of 1–3 μg of Cd [1,4,7]. The kidney is the main organ affected by chronic Cd exposure. Ninety percent of filtered Cd is reabsorbed in the proximal tubule particularly in the S1 and S2 segments [8,9]. It has been reported that Cd enters proximal tubular cells by different routes: endocytosis (Cd conjugated with metallothionein or low molecular thiols) and transporters such as zinc transporter 1(ZnT1), ATP-binding cassette protein (ABC protein; located at basolateral membrane), ZrT/Irt-like protein (ZIP), Divalent Metal Transporter 1 (DMT1), and Na^+^-amino acid co-transporters [9,10]. Inside the cell, Cd induces proteins such as metallothionein 1/2 (MT1/2) and interacts with their sulfhydryl groups, altering protein function [11,12]. Main nephrotoxic symptoms of Cd exposure are proteinuria, glycosuria, aminoaciduria, and phosphaturia similar to Fanconi’s syndrome [11,13]. The Agency for Toxic Substances and Disease Registry (ATSDR) has determined a dose of 0.1 μg Cd/kg/day as Minimal Risk level (MRL) based on low molecular weight proteinuria (LWP) during a chronic-oral exposure [1]. Different studies have been designed to elucidate the mechanisms by which Cd induces proteinuria [14-16]. However, it has been reported that Cd toxicity depends on dose, route of administration and time of exposure. The glomerulus retains large proteins (>67 kDa), but low weight proteins pass freely through the glomerular barrier and are reabsorbed along the proximal tubule. Normally, a healthy adult excretes from 40 to 80 mg of protein per day, from which 10 to 15 mg is albumin [17,18]. Filtrated albumin is reabsorbed in the proximal tubule; therefore albuminuria may result from an over-filtration of plasma proteins, glomerular or tubular dysfunction [17,19,20]. Protein reabsorption at the proximal tubule is accomplished by receptor-mediated endocytosis. Two receptors responsible for endocytosis, megalin and cubilin, have been described as major players in this phenomena [21,22]. Megalin is a glycoprotein of 600 KDa, member of the low-density lipoprotein receptor (LDLR) family. Megalin is a multiligand receptor with a large NH_2_-terminal extracellular domain rich in cysteine, a single transmembrane domain and a COOH-terminal short cytoplasmic domain with NPXY motifs that are responsible for internalization mediated by clathrin [22-24]. On the other hand, cubilin is a peripheral membrane protein of 460 KDa, without transmembrane and cytoplasmic domains; cubilin contains 27 COOH-terminal CUB domains (complement subcomponents C1r/C1s, Uegf) which are ligand binding. Cubilin has different ligands, including megalin. In the proximal tubule cells, cubilin is co-expressed with megalin, and interacts with it, forming a complex to be internalized [25]. A decrease in the expression of megalin receptor favors the presence of proteins into urine (as captesin B, RPB and albumin) [21] and cubilin deficiency leads to an increase in albumin excretion [26]. Endocytosis along the proximal tubule is regulated by different factors, including angiotensin II (Ang II), an active vasopeptide of the renin-angiotensin system (RAS). RAS is an hormonal system that begins with renin secretion; this enzyme acts upon its substrate angiotensinogen, resulting in the formation of angiotensin I (Ang I) which in turn is hydrolyzed and converted to Ang II by the angiotensin converting enzyme (ACE) [27,28]. It has been suggested that Ang II can downregulate megalin and the treatment with RAS inhibitors or AT1 receptor blockers decreases proteinuria and protects kidney function independent of the effect of drugs on blood pressure [29,30]. In diabetic rats the infusion of Ang II decreases megalin expression and albumin reabsorption, but AT1 receptor blockers may restore this effect [31]. Hosojima et al. (2009) proposed that Ang II negatively regulates megalin expression at both mRNA and proteins levels, through the AT1 receptor and ERK pathway activation [32]. On the other hand, there is evidence that the RAS is implicated in the toxicity of cadmium [33-35]. In the present study, we evaluated the role of the megalin-cubilin complex and AT1 receptor on tubular endocytosis of albumin during a subchronic exposure to Cd in rats.

## Methods

### Animals and treatment

Female Wistar rats (180–220 g bw) were used. Animals were housed with 12/12-h light/dark cycles at 22 ± 1°C, 50 ± 5% humidity and received standard chow (PMI, 5008, Purina, Alief City, TX) and purified water *ad libitum*. The use of animals was in accordance with the Institute for Laboratory Animal Research (ILAR) Guide for Care and Use of Laboratory Animals and was approved by the Institutional Ethical Committee at Cinvestav, CICUAL [Comité Interno para el Cuidado y Uso de los Animales de Laboratorio; Number 391–07].

Rats were divided into four groups: 1) Control (CT): rats were administered with water by gavage for eight weeks. 2) LOS: from the fifth week on, the rats were administered with Losartan, an AT1 antagonist (Cozaar, 50 mg, Merck Sharp and Dohme de México S.A. de C.V.) (10 mg/kg/day, by gavage). 3) Cd: rats were administered with cadmium chloride (CAS No. 10108-64-2, Sigma Aldrich) (3 mg/kg/day, by gavage) for 8 weeks (weeks 0–8). 4) Cd + LOS: rats were administered with Cd for 8 weeks and LOS from weeks 5 to 8. One day after the end of treatment, rats were housed individually in metabolic cages to collect 16 h urine for the measurements of microalbuminuria, creatinine, N-acetyl-β-D-glucosaminidase (NAG) and glucose. After urine collection, animals were sacrificed with an overdose of Nembutal (pentobarbital sodium, 50 mg/100 g bw, i.p.). The kidneys were removed and washed in cold PBS. A half of the right kidney was used to determine cadmium content and a second half was used to extract RNA for RT-PCR. A half of the left kidney was frozen at -70°C using 2-methylbutane as cryoprotector for immunofluorescence assays and a second half was used to extract proteins for Western blot and oxidative stress measurements.

### Urine and plasma measurements

Urine samples were centrifuged for 10 min at 1000 × g, and aliquots were separated. Blood samples were obtained by cardiac puncture and centrifuged for 15 min at 2000 × g to obtain plasma. Creatinine was measured in plasma and urine using the Jaffé method (CR510, Randox Laboratories, San Diego, CA) and glucose was measured in urine by the hexokinase method (GL161, Randox Laboratories, San Diego, CA). Microalbuminuria was measured using the HemoCue Albumin 201 system. Urinary NAG was determined at 580 nm by using a colorimetric assay (Cat. No. 875 406; Roche diagnostic).

### Creatinine clearance was calculated using the standard equation

$$C_{\mathrm{cr}}=\left(U_{\mathrm{cr}}/P_{\mathrm{cr}}\right)x\;\mathrm{Jv}$$

where C_cr_ is the value of creatinine clearance (μL/min/100 g bw), U_cr_, and P_cr_ are the concentration values (mg/dL) of creatinine in urine and plasma, respectively, and Jv is the urinary flow rate (μL/min/100 g bw).

### Measurement of renal cadmium content

The technique used to quantify Cd content was described previously by the Environmental Protection Agency (EPA) [36]. Kidney samples were weighed and digested in a solution (2:1) of nitric acid (9601–02, Reactive Grade, J.T. Baker, 69.9% of purity) and hydrochloric acid (9535–02, Reactive Grade, J.T. Baker, 37.1% of purity) plus 3 mL of hydrogen peroxide (2186, Reactive Grade, J.T. Baker, 30% of purity) overnight. Afterwards, samples were heated to a temperature of 80-90°C until full organic matter digestion was observed by the release of nitrous fumes (yellow-orange). In order to remove any organic residue, 3 mL of hydrogen peroxide were added to samples for 5 min, leaving the mixture to react at 80-90°C until the solution turned transparent. At the end of this period, the solution was filtered and diluted to 10 mL with deionized water. In the final solution, Cd concentration was quantified by a standard curve using atomic absorption direct aspiration flame (Perkin Elmer, AAnalyst 100), with detection limit of 0.008 mg/L. The following calculation was performed to express the concentration in mg/g tissue:

$$\begin{array}{ll} \mathrm{Cd}\;\left(\mathrm{mg}/g\right)= & \left[\mathrm{Cd}\;\left(\mathrm{mg}/L\right)\right]*\text{Volume}\;\mathrm{of}\;\text{dilution}\;\left(L\right)\\ & \;/\;\text{weight}\;\mathrm{of}\;\text{sample}\;\left(g\right) \end{array}$$

### Lipid peroxidation determination

Renal cortex was homogenized in phosphate buffer (50 mM, pH 7.4) containing 10 μL of butylated hydroxytoluene (0.5 M) in acetonitrile. Subsequently, the homogenate was centrifuged for 10 min (3000 x g at 4°C). Protein concentrations of the supernatants were determined using the Lowry assay. The supernatants were mixed with a solution (1:3) of 1-methyl-2-phenylindole in a mixture of acetonitrile methanol (3:1), reactions were started adding 37% HCl. Later, samples were incubated for 40 min at 40°C. Lipid peroxidation was evaluated by measuring 4-hydroxynonenal (4-HNE) and malondialdehyde (MDA) using a standard curve of tetramethoxypropane at 586 nm [37,38].

### NAD(P)H oxidase activity determination

Renal cortex was homogenized in phosphate buffer (50 mM, pH 7.4; w/v 1:10), containing 1 mM EDTA, 0.1% triton-X100 and protease inhibitors. Subsequently the homogenate was centrifuged for 10 min (6000 x g at 4°C). Supernatants were incubated with 0.02 mM dihydroethidium (DHE), 0.05 mg/ml salmon testes DNA, and the corresponding substrate for NAD(P)H oxidase in a dark plate away from direct light and at 37°C for 30 min. DHE is oxidized to ethidium (Eth) and is used as a marker of superoxide (O_2_^•¯^) formation. Eth-DNA fluorescence was measured at an excitation wavelength of 480 nm and an emission of 610 nm by using a fluorescence multimode microplate reader (Synergy HT; Biotek) [39]. 0.1 mM of NADH was used as a substrate in the reaction mixture. To further confirm that the activity of NAD(P)H oxidase contributes to the production of O_2_^•¯^; diphenylene iodonium (DPI, 0.1 mM) a NAD(P)H oxidase inhibitor, was used. The fluorescent signal of each sample was expressed as DHE fluorescence relative to the control. Protein concentrations of the supernatants were determined using the Lowry assay.

### Immunofluorescence

Kidneys were sectioned in a Leica CM1100 cryostat (sections of 8 μm) and the slices were transferred on gelatin-coated slides. The slices were fixed for 10 min with methanol at -20°C; thereafter the slices were washed with PBS (with 1 mM calcium) and then permeabilized with 0.2% Triton-X100 for 5 min at room temperature, and afterwards, washed twice with PBS. After the blocking them for 1 h at room temperature with 1% (w/v) IgG-free-albumin, the slices were incubated overnight at 4°C with primary polyclonal goat anti-megalin antibody (sc-16478, Santa Cruz Biotechnology, Inc., dilution 1:50), polyclonal goat anti-cubilin antibody (sc-20609, Santa Cruz Biotechnology, Inc., dilution 1:50), polyclonal goat anti Kim-1 antibody (Cat. No. AF3689, R&D System, dilution 1:500) or monoclonal mouse anti-dipeptidyl-peptidase (IV) antibody (MCA924, Serotec Ltd., Kidlington, Oxford, OK, dilution 1:500 ). After three washes for 5 min with PBS at room temperature, the secondary antibody Alexa-Fluor 594 donkey anti goat (A11058, Invitrogen, dilution 1:300), Alexa-Fluor 488 donkey anti goat (A11055, Invitrogen, dilution 1:500) or Alexa-Fluor 594 donkey anti mouse (A21203, Invitrogen, dilution 1:500) was added for 2 h at room temperature. Later, the slices were washed three times with PBS, and mounted with Vectashield H-1400 (Vector laboratories Inc. Burlingame, CA). The immunofluorescence was examined using a multiphotonic confocal microscope (Leica TCSSP5 MO PANDEM) and analyzed with Leica Microsystem LAS AF Lite software. Megalin and cubilin fluorescence intensity was quantified in three images from three different rats per group.

### Western-blot

The renal cortex was homogenized in cold buffer A (250 mM sucrose, 5 mM EDTA, 10 mM HEPES, protease inhibitors at pH 7.8), and then centrifuged at 4°C for 15 min 13000 x g, the pellet was resuspended on ice cold buffer B (2 mM CaCl_2_, 1 mM MgCl_2_, 10 mM HEPES, 140 mM NaCl, protease inhibitors, and 1% Triton X-100 at pH 7.8). Afterwards, the samples were centrifuged at 4°C for 30 min at 13000 x g. Protein concentrations of the supernatants were determined using Pierce BCA protein assay (No. 23225, Thermo Scientific). The supernatants were electrophoresed on 12% SDS-PAGE polyacrylamide and the proteins were transferred to nitrocellulose membranes (Bio-Rad Laboratories). Membranes were blocked for 1 h with 5% low-fat dry milk, next the blots were incubated with primary rabbit anti-AT1 antibody (sc-579, Santa Cruz Biotechnology, Inc.), diluted 1:200 in 10 mM PBS (Cat. No. 21300–058, Gibco by Life Technologies) containing 0.1% tween overnight at 4°C. Blots were washed three times for 10 min in 10 mM PBS containing 0.1% tween and incubated for 1 h with the secondary antibody (goat anti-rabbit IgG 1:10000, sc-2004 Santa Cruz Biotechnology, Inc.) at room temperature. After three washes for 10 min in 10 mM PBS containing 0.1% tween, the proteins were detected by a chemiluminescence assay (RPN 2109, Amersham).

### Real-time PCR

The RNA was extracted withTrizol protocol, using 100 mg of renal cortex; and the cDNA was synthetized with the ImProm-II Reverse Transcription system (A3800, Promega Corporation). The megalin and cubilin genes were analyzed by quantitative RT-PCR using the TaqMan system in a 7500 real time PCR system (Applied Biosystems). Fluorescence for each cycle was analyzed quantitatively, and gene expression was normalized for the expression of the housekeeping gene GAPDH (Part Number 4352338E, Applied Biosystems). The forward and reverse primers for megalin are listed in Table 1, and for the cubilin gene, a commercial TaqMan gene expression assay was used (Part Number 4331182, Applied Biosystems).

**Table 1:** List of primers used for megalin gene

**Rat megalin gen**	**Accession no. L34049**
Forward primer	AATGCGGCAGTGGGAATTTT
Reverse primer	ACACCCAGGAGCTAGGGAT
Probe	/5′6FAM/TGGCTGTCCTCCCAGGTCCTGC/3′TAMRA

### Statistical analyses

Data were analyzed by one-way ANOVA or Kruskal-Wallis test using GraphPad Prims 5 software (Version 5.00). KS normality test and Bonferroni’s or Dunn’s poshoc test were used. P < 0.05 was considered statistically significant. The data are expressed as means ± SEM.

## Results

### Effect of Cd on body weight, water intake and renal content

The first aim of this study was to explore the effect of Cd treatment on physiological parameters such as body weight, water intake and renal accumulation of this metal on the different treated groups at the end of the exposure period. Table 2 shows, no alterations on body weight in the different experimental groups. Interestingly, Cd + LOS group showed a significant increase in water intake (approximately 2-fold) when compared with CT, LOS and Cd groups.

**Table 2:** Effect of subchronic exposure to cadmium on body weight, water intake and renal cadmium content

**Experimental group**	**CT**	**LOS**	**Cd**	**Cd + LOS**
Body weight (g)	263 ± 19	256 ± 22	263 ± 21	256 ± 17
Water intake (ml/100 g/16 h)	8.33 ± 2.94	11.38 ± 6.33	10.20 ± 3.84	20.20 ± 13.75^a,b,c^
Renal Cd levels (μg/g wet tissue)	0.08 ± 0.00	0.08 ± 0.00	7.6 ± 1.12^d,e^	7.6 ± 0.97^d,e^

*LOS*, losartan. Data represent the means ± SEM, n = 9–10. ^a^P < 0.05 vs CT, ^b^P < 0.05 vs LOS, ^c^P < 0.05 vs Cd, ^d^P < 0.001 vs CT, ^e^P < 0.001 vs LOS.

As expected, gavage administration of Cd increased renal Cd content, which was significant in both, Cd and Cd + LOS groups (7.6 μg/g kidney) compared to CT and LOS groups (0.087 μg/g kidney) (Table 2).

### Effect of Cd on urinary flow rate, plasma creatinine and creatinine clearance

**Figure 1:**
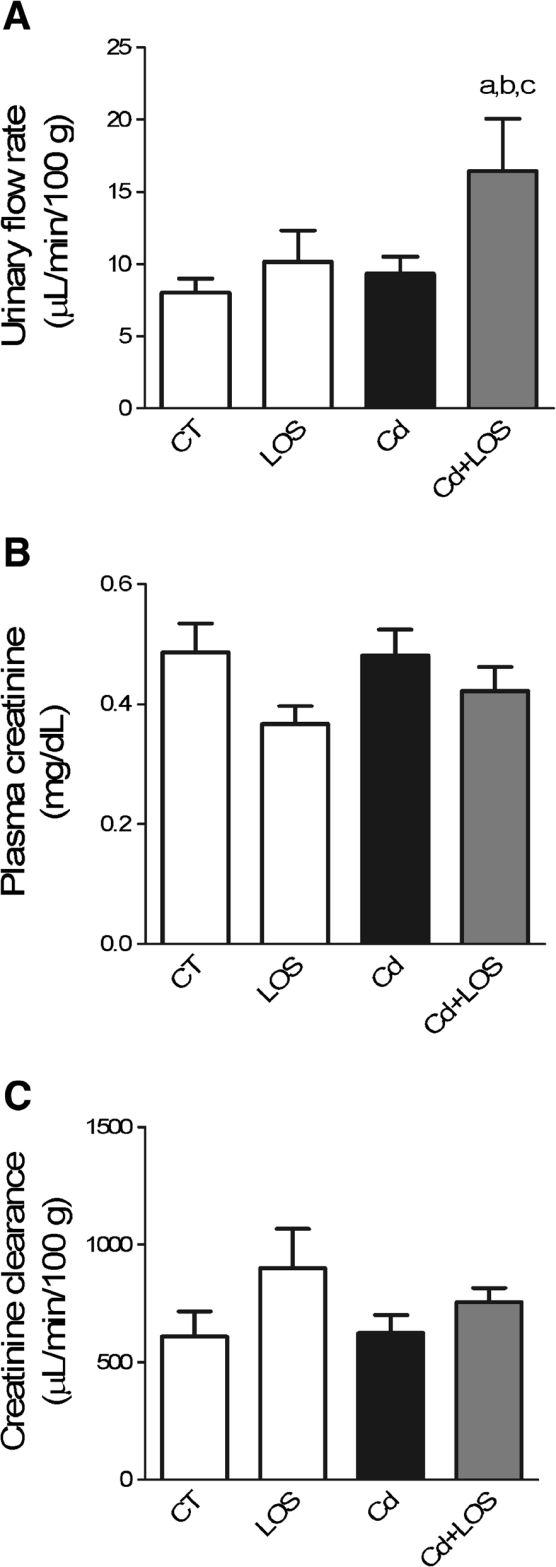
**Effect of subchronic exposure to cadmium on renal function parameters.** Urinary flow rate **(A)**, increased in cadmium + losartan (Cd + LOS) group compared to control (CT), losartan (LOS), and cadmium (Cd) groups. Plasma creatinine **(B)**, and creatinine clearance **(C)** did not change in the all experimental groups studied. Data are means ± SEM, n = 9–10 rats per group. ^a^P < 0.05 vs CT, ^b^P < 0.05 vs LOS, ^c^P < 0.05 vs Cd. Urinary flow rate was analyzed with one way ANOVA and Bonferroni’s multiple comparison test; plasma creatinine and creatinine clearance were analyzed with Kruskal-Wallis and Dunn’s multiple comparison test.

To determine whether Cd alters renal function in our experimental conditions, we measured urinary flow rate (Jv) and estimated glomerular filtration rate (eGFR) determined by creatinine clearance (Figure 1).

Rats treated with Cd + LOS showed increased urinary flow rate compared with CT (p < 0.05), LOS (p < 0.05), and Cd (p < 0.05) groups (Figure 1A). Meanwhile, no changes were found in plasma creatinine and creatinine clearance in the different treated groups (Figure1B and C) our results indicate that none of the treatments affected glomerular function.

### Effect of Cd on urinary glucose concentration, urinary NAG, microalbuminuria and Kim-1 expression

**Figure 2:**
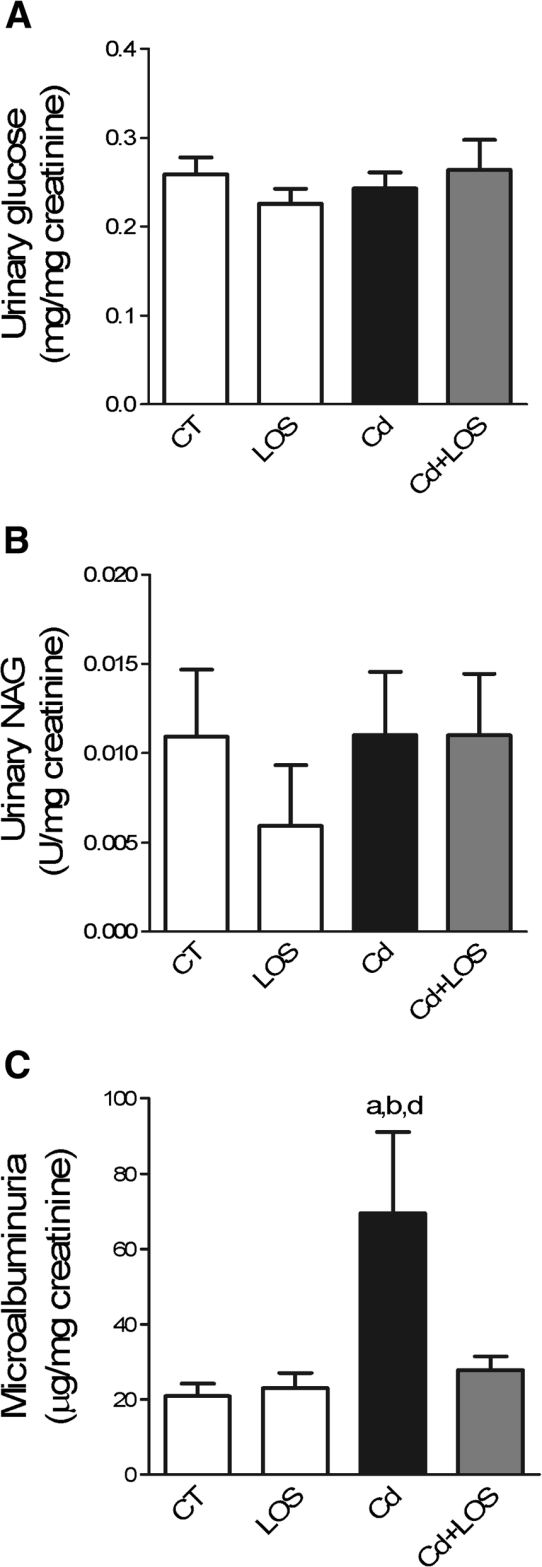
**Effects of subchronic exposure to cadmium on markers of proximal tubule damage.** Urinary glucose **(A)**, N-acetyl-β-D-glucosaminidase (NAG) **(B)**, and microalbuminuria **(C)** in control (CT), losartan (LOS), cadmium (Cd) and cadmium + losartan (Cd + LOS) groups. Co-treatment with LOS (Cd + LOS group) decreased microalbuminuria induced by cadmium (Cd group). Urinary glucose **(A)** and NAG **(B)** did not change in the all experimental groups. Data are means ± SEM. n = 9–10 rats per group. ^a^P < 0.05 vs CT, ^b^P < 0.05 vs LOS, ^d^P < 0.05 vs Cd + LOS. Urinary glucose and microalbuminuria were analyzed with one way ANOVA and Bonferroni’s multiple comparison tests; urinary NAG was analyzed with Kruskal-Wallis and Dunn’s multiple comparison tests.

In order to explore proximal tubular function, urinary concentrations of glucose, and NAG, and microalbuminuria were determined (Figure 2).

**Figure 3:**
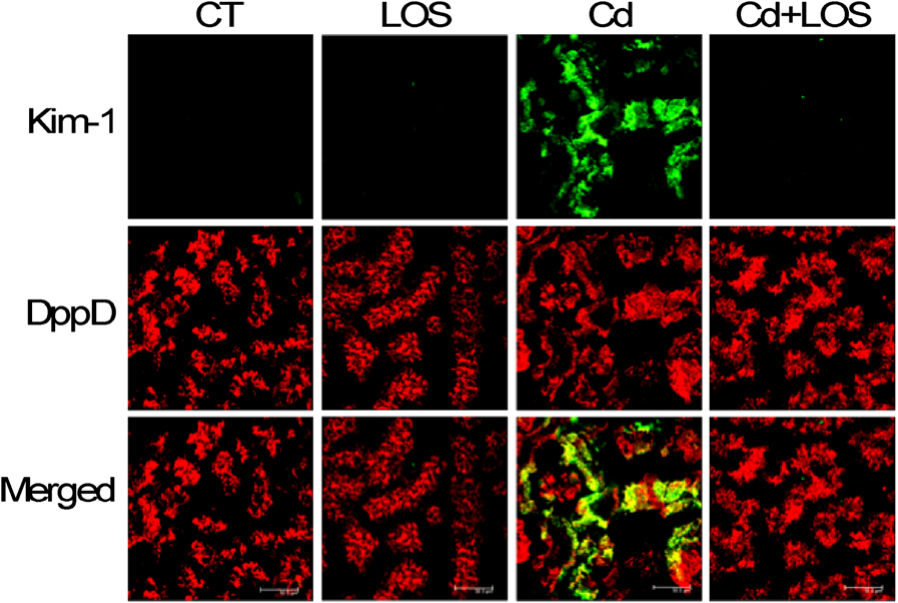
**Effect of subchronic exposure to cadmium on Kim-1 protein expression.** Representative confocal images of: Kim-1 with dipeptidyl peptidase 4 (DppD), as tubule proximal marker. No expression of Kim-1 was observed in the control (CT), losartan (LOS), and cadmium + losartan (Cd + LOS) groups, it was observed only in the cadmium group (Cd). n = 3 rats per group.

No changes were found in urinary excretion of glucose and NAG in the different treated groups. However, microalbuminuria was significantly increased in Cd treated animals when compared with control groups (CT and LOS treated rats) (p < 0.05). Kim-1 was evaluated as an early biomarker of proximal tubule injury showing a clear expression only in rat kidneys of the Cd group (Figure 3). The co-administration with LOS reduced Kim-1 expression and decreased the microalbuminuria when it was compared to Cd treated rats (Cd vs. Cd + LOS; p < 0.05), indicating a possible relationship between the impaired endocytosis and injury induced by Cd and the blockade of AT1 receptor with LOS.

### Effect of Cd on kidney oxidative stress

Increased oxidative stress has been demonstrated to be involved in Cd toxicity and a possible relationship with proteinuria has been reported. To evaluate the redox status in the exposed animals, we measured malondialdehyde and 4-hydroxynonenal content in renal cortex (both used as markers of lipid peroxidation) (Table 3). No changes were found in lipid peroxidation in the four different treated groups. In order to explore the production of a specific reactive oxygen specie (ROS), we measured superoxide (O_2_^•^_¯_) production by assessing NAD(P)H oxidase activity, since it has been reported that the overactivation of this enzyme constitutes a major source of oxidative stress in the kidney [40]. Also, it has been documented that NAD(P)H oxidase is activated via Ang II through the AT1 receptor [41]. On Table 3, it can be observed that the rats treated with Cd + LOS showed a significantly decrease activity of NAD(P)H oxidase compared to the CT group (P < 0.05). Based on the above-described results, it was concluded that under our experimental conditions Cd-treatment at 8 weeks did not induce oxidative stress imbalance.

**Table 3:** Effect of subchronic exposure to cadmium on lipid peroxidation and NAD(P)H oxidase activity

**Experimental group**	**CT**	**LOS**	**Cd**	**Cd + LOS**
Lipid peroxidation (nmol MDA + 4-HNE/mg)	2.58 ± 0.42	1.26 ± 0.39	1.52 ± 0.26	2.08 ± 0.38
NAD(P)H oxidase activity (FU/mg)	8005 ± 962	5972 ± 1560	5415 ± 1493	3707 ± 577^a^

*LOS*, losartan; *MDA*, malondialdehyde; *4-HNE*, hydroxynonenal; *FU*, fluorescence unit. Data represent the means ± SEM. n = 5. ^a^P < 0.05 vs CT.

### Effect of Cd on megalin expression

**Figure 4:**
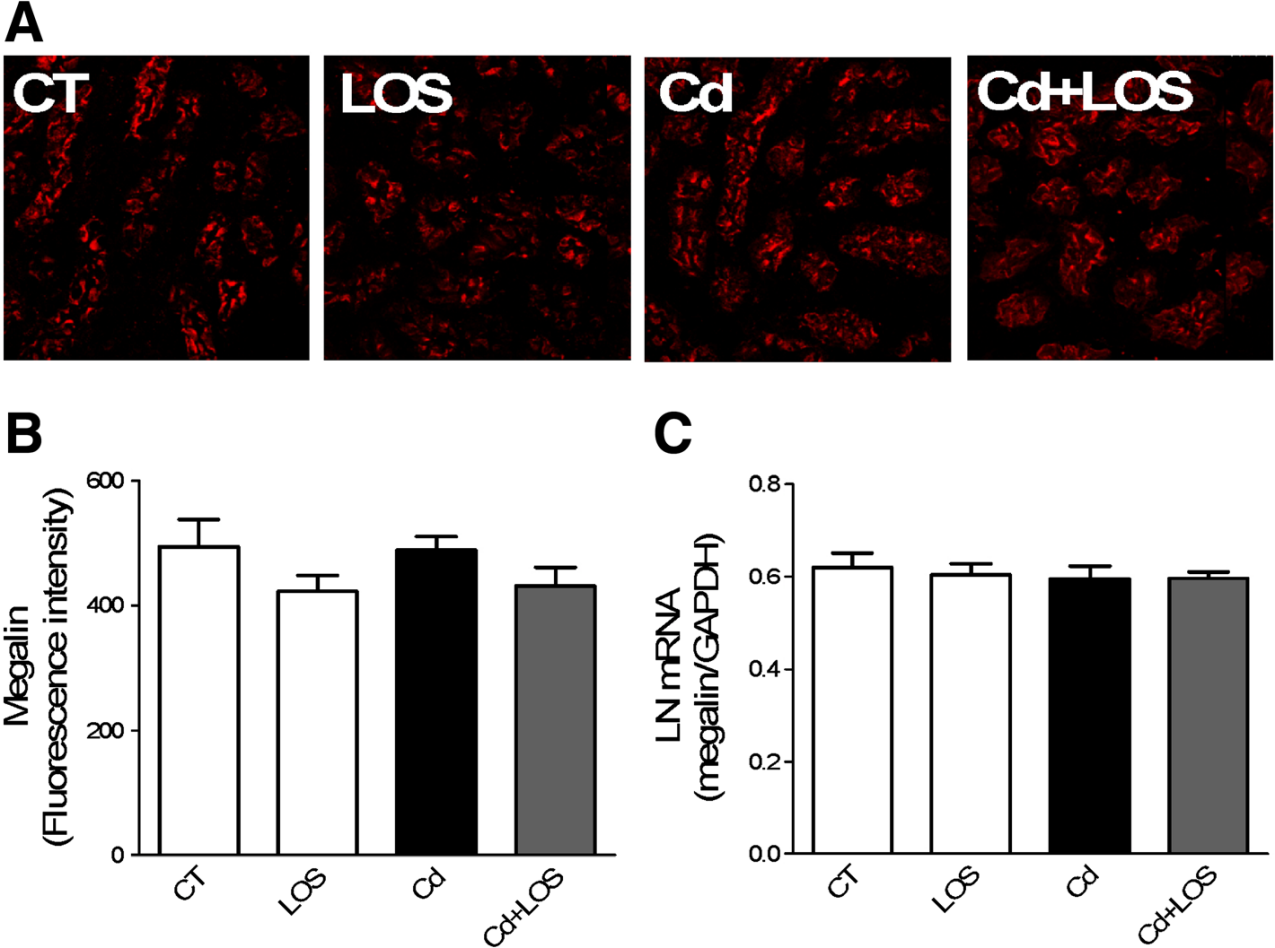
**Effect of subchronic exposure to cadmium on megalin’s mRNA and protein expression.** Megalin expression did not change in losartan (LOS), cadmium (Cd) and cadmium + losartan (Cd + LOS) groups compared to control group (CT), as observed by immunofluorescence micrographs **(A)**. Quantification of fluorescence intensity is shown in panel **(B)**. mRNA levels of megalin did not change in the four experimental groups **(C)**. Data are expressed as LN mRNA of means ± SEM, n = 3-10 rats per group. Fluorescence intensity and mRNA were analyzed with one way ANOVA and Bonferroni’s multiple comparison tests.

**Figure 5:**
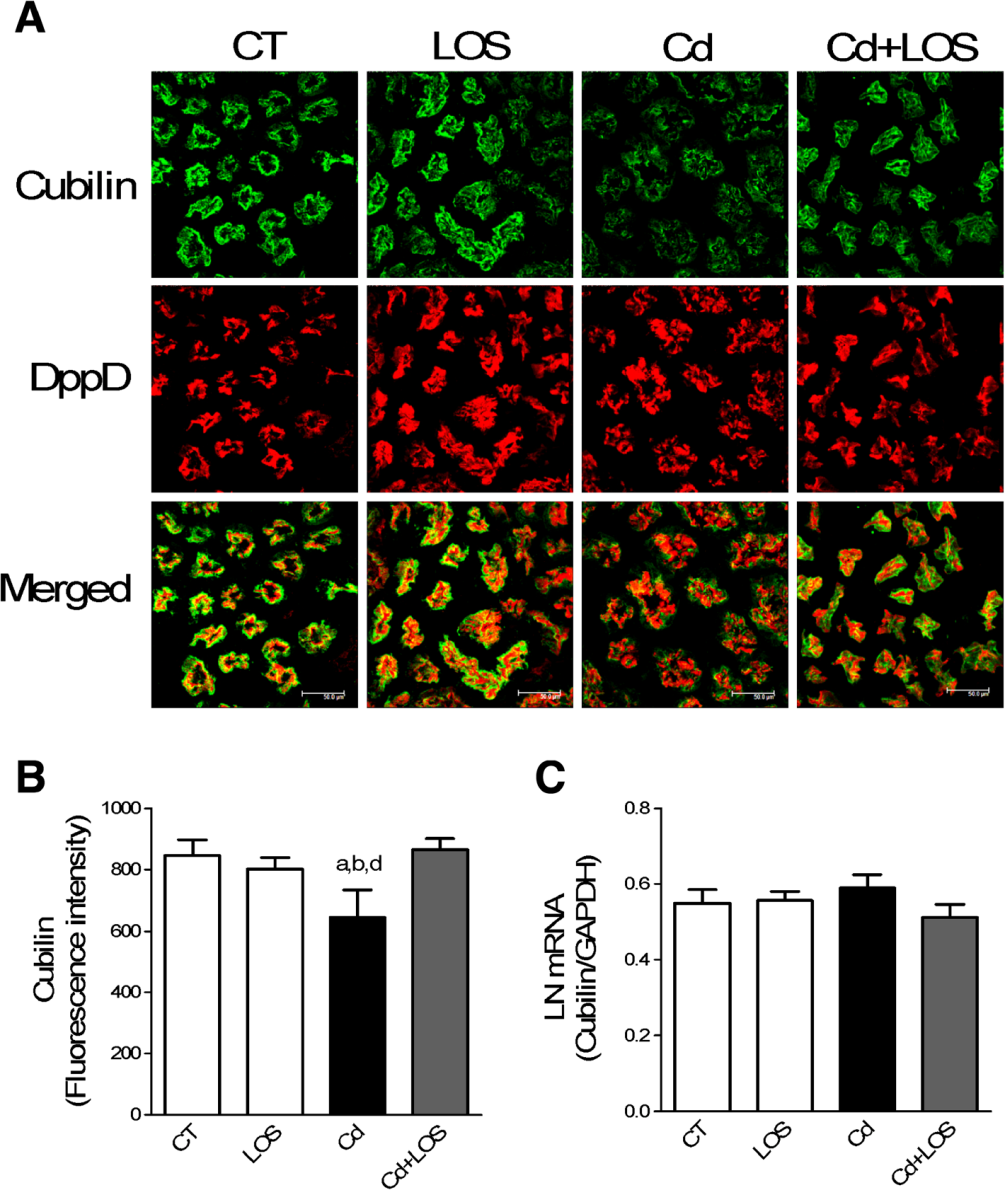
**Effect of subchronic exposure to cadmium on cubilin’s mRNA and protein expression.** Cubilin expression did not change in losartan (LOS), and cadmium + losartan (Cd + LOS) groups compared to control group (CT), but was decreased in cadmium (Cd) group as observed by immunofluorescence micrographs **(A)**. Quantification of fluorescence intensity is shown in panel **(B)**. mRNA levels of cubilin did not change in the four experimental groups **(C)**. Data are expressed as LN mRNA of means or fluorescence intensity ± SEM, n = 3-10 rats per group. Statistical analysis was performed with Kruskal-Wallis and Dunn’s multiple comparison tests for fluorescence intensity, and one way ANOVA and Bonferroni’s multiple comparison tests for mRNA levels.

Studies have demonstrated that megalin and cubilin receptors internalize albumin and LWP in tubular proximal cells [25]. To further determine whether megalin and cubilin could be involved in the decreased endocytosis (suggested by microalbuminuria observed in Cd-treated rats); it was investigated whether Cd-treatment affected the localization and expression of megalin and cubilin by using confocal microscopy (Figure 4A and Figure 5A). The quantification of the fluorescence intensity was also evaluated (Figure 4B and Figure 5B). As shown in Figure 4A, megalin label was observed in the apical membrane of epithelial proximal cells in the four different groups and no changes were found along the experimental groups. Consistently the quantification of fluorescence intensity showed no changes in the different groups (Figure 4B). Furthermore, Cd, LOS or Cd + LOS treatments did not affect mRNA levels of megalin (Figure 4C). On the other hand, cubilin label, localized in the apical membrane of epithelial proximal cells was decreased in the Cd exposed group (Figure 5A) as confirmed by the quantification of fluorescence intensity showing statistically significant differences in the Cd group compared with the others groups (Figure 5B). Interestingly mRNA levels of cubilin were not modified by Cd exposure (Figure 5C). These findings suggest that cubilin might be participating in the increased microalbuminuria observed in the Cd-group whereas megalin might not.

### Effect of Cd on AT1 receptor expression

**Figure 6:**
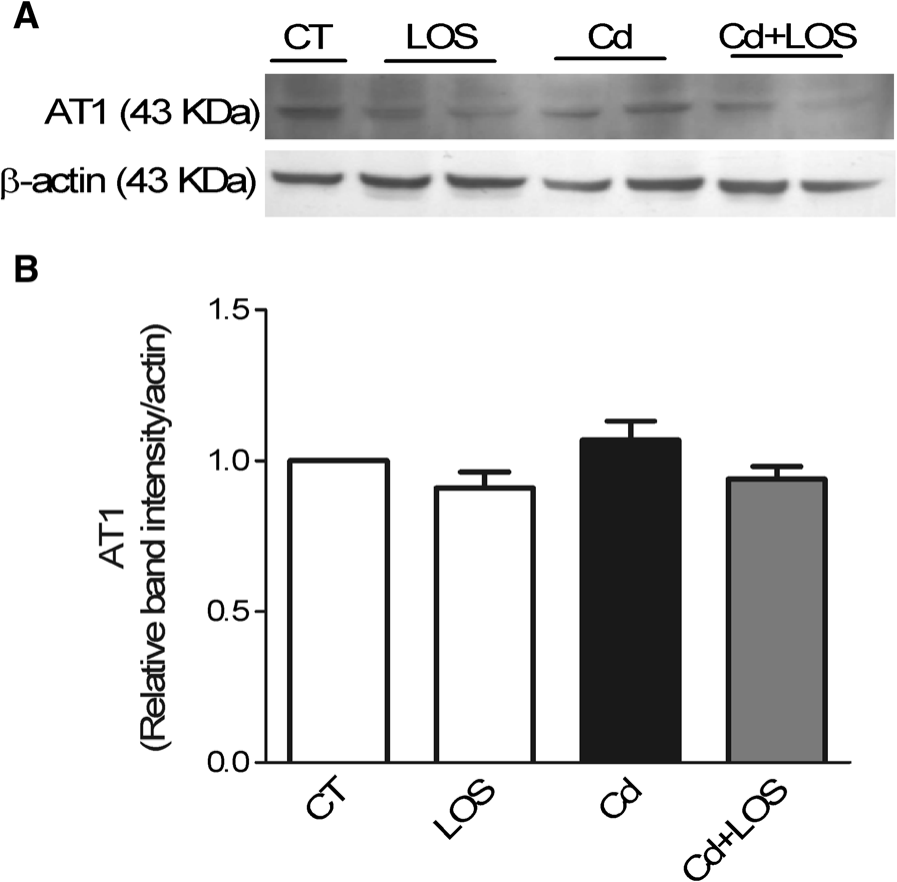
**Effect of subchronic exposure to cadmium on angiotensin II type 1 (AT1) receptor expression.** AT1 receptor expression did not change in losartan (LOS), cadmium (Cd) and cadmium + losartan (Cd + LOS) groups compared to control group (CT), **(A)**. Densitometry is shown in panel B. Representative image of one of three independent experiments is shown **(A)**. Relative band intensity was normalized with actin level **(B)**. Data are means ± SEM, n = 3. Statistical analysis was performed with Kruskal-Wallis and Dunn’s multiple comparison tests.

Since LOS (an AT1 receptor antagonist) treatment decreased the impaired endocytosis induced by Cd, it was decided to analyse the expression levels of AT1 in the different treated groups. As it can be seen in Figure 6, no changes were found in protein levels of AT1 receptor in the four groups studied (Figure 6A and B).

## Discussion

Kidney is the main target organ of Cd exposure. Chronic exposure to low levels of Cd produces urinary waste of LWP, suggesting an alteration of endocytosis in the proximal tubule. However, the mechanism responsible of Cd-induced proteinuria is not fully understood. In normal physiological conditions, LWP can be filtered through the glomerulus, but they are reabsorbed by endocytosis in the proximal tubule. The megalin-cubilin complex is responsible for the endocytosis of proteins in the renal proximal tubule [25]. On the other hand, it has been observed that the use of RAS inhibitors prevents proteinuria in diabetic patients and rats [29-31,42]. Therefore, the aim of this study was to evaluate the role of megalin-cubilin complex and AT1 receptor on impaired tubular endocytosis of albumin during a subchronic exposure to Cd. In this study, we decided to administrate Cd by gavage, for two main reasons: 1) to control the administered dose, and 2) oral route reflects the dietary exposure that is the most frequent route in the non-smoking population [4-6]. To better assess cellular mechanisms involved in Cd-nephrotoxicity, we decided to use a low dose that would not induce extensive damage in proximal tubules. Herein, the treatment with 3 mg Cd/kg/day by gavage induced microalbuminuria. This effect was counteracted by treatment with LOS, which suggests the involvement of AngII and AT1 receptor in microalbuminuria induced by Cd. Chronic exposure to Cd induced proteinuria; this effect has been observed in occupationally exposed populations [7,43,44] and experimental studies have tried to elucidate the mechanism of Cd induced decrease in protein endocytosis. Choi et al. (1999) observed that in Opossum Kidney epithelial (OK) cells, endocytosis of albumin was inhibited by exposure to Cd, but this effect was independent from substrate affinity or direct interaction of Cd on the endocytic receptor [16]. The albumin endocytosis occurs via a receptor-mediated mechanism. In the proximal tubule, there are two principal endocytic receptors: megalin and cubilin. Megalin is a large protein ≈ 600 kDa, and it is a receptor constitutively concentrated in proximal tubular brush-border, coated pits, and vesicles. This receptor has a cytoplasmic tail that contains an internalization motif (two NPXY and a NPXY-like); which mediates binding to adapter proteins and the formation of the endocytic vesicle (internalization mediated by clathrin) [20,45]. Cubilin is a peripheral membrane glycoprotein (≈460 kDa), without transmembrane and intracellular domains, and is co-expressed with megalin in the proximal tubule. Cubilin also binds to proteins such as albumin, and is considered essential for albumin reabsorption [26]. However, cubilin needs to bind megalin to be internalized. Although, cubilin also binds amnionless (AMN); a protein that colocalizes with it, and is important for the traffic of cubilin to the apical membrane [25]. Moreover, proteinuria induced by Cd exposure could be due to a) a decreased expression of membrane receptors involved in endocytosis, or b) inhibition of endocytosis via the alteration of one of the stages of this mechanism (internalization, vesicle recycling, or acidification of endo/lysosomes). Gena et al. (2010) observed that Cd exposure decreased megalin expression in Lilly Laboratory Cell Porcine Kidney cells (LLC-PK1) which was associated with impaired albumin endocytosis [14]. In our study, we analyzed the expression of both, protein and mRNA levels of megalin. In contrast with previous studies, no changes were observed in mRNA and protein levels of megalin in Cd-treated rats. Interestingly, expression of cubilin was decreased by Cd exposure without changes in mRNA levels; suggesting that Cd might induce a post-transcriptional modification of cubilin. After albumin filtration, this protein is reabsorbed along proximal tubule cells by endocytosis. Cubilin has been considered the most important receptor in the albumin reabsorption [26,46]. In our study, microalbuminuria induced by Cd was attenuated with LOS, also cubilin levels were recovered in LOS-treated rats, indicating a possible relationship between cubilin receptor decrease and microalbuminuria. This data is in agreement with the findings reported by Oroszlán et al. (2010), where the treatment with ramipril (an ACE inhibitor) and LOS prevented downregulation of megalin and cubilin receptors induced by proliferation signal inhibitors (PSIs) [47]. Increased urinary albumin can be due to two factors: increased glomerular filtration rate and/or a decreased tubular reabsorption. In our model, glomerular function was evaluated by measuring plasma creatinine and creatinine clearance. No changes were found in both parameters evaluated in the four groups studied. Thus, glomerular function was not apparently affected after Cd exposure. In addition, urinary levels of glucose and NAG were unchanged by exposure to Cd, suggesting the absence of severe proximal tubule damage. However, Kim-1 expression was increased after Cd exposure evidencing a tubular injury, which is consistent with the observations made by Prozialeck et al. (2007) where Kim-1 was proposed as an early biomarker of Cd nephrotoxicity [48]. In this study, LOS administration decreased Kim-1 expression and microalbuminuria induced by Cd.

In addition to megalin and cubilin receptors; endocytosis involves multiple cellular components such as the endosome. The endosome needs some other molecular components for its optimal acidification: Na+/H + exchanger (NHE3), vacuolar proton–ATPase (v-H + -ATPase), and chloride channel 5 (ClC5) [45]. Absence of ClC5 is related to a dysfunctional endosomal acidification and therefore alterations in the reabsorption and the endocytosis, leading to a renal tubular disorder called Dent’s disease. In an *in vitro* model, LLC-PK1 cells incubated with 10 μM of Cd, ClC5 expression was decreased after 3 h of exposure [14]. In *in vivo* models, some authors have observed that Cd exposure inhibits both v-H^+^-ATPase [49] and NHE3 [50]. However, in these studies, rats received daily subcutaneous injections of Cd (2 mg Cd/kg/day, 14 days or 3 weeks, respectively) reaching Cd concentrations in the kidney from 156 to 265 μg/g wet tissue. It is possible that in the above mentioned studies animals showed glomerular damage, due to, in both experiments, urinary flow and total protein excretion increased after Cd exposure.

To explore the mechanism of Cd toxicity, we evaluated oxidative stress by assessing lipid peroxidation. In our model, we did not observe changes on lipid peroxidation in any the groups studied. However, Wang et al. (2009) reported, that one of the mechanisms by which Cd induced nephrotoxicity is by reactive oxygen species (ROS) overproduction, inferred from the use of an antioxidant agent (N-acetyl cysteine) that protected against Cd-induced proteinuria [51]. Many other studies indicate that Cd promotes oxidative stress imbalance [52-54]. It is possible that we have not detected oxidative stress injury; may be due to the low amount of Cd and length of exposure (3 mg/kg/day for 8 weeks). Absorption of Cd on gastrointestinal tract is low (≈5-10%), in our model, a low concentration of Cd reached the kidneys (7.6 μg/g wet tissue), and it is possible that renal cells were still able to respond adequately to the oxidative insult. Thijssen et al. (2007) report that Cd exposure may trigger a biphasic defense response in the kidney, and could lead to adaptation and survival [55] maybe by induction of Nuclear factor erythroid 2 related factor 2 (Nrf2), this transcription factor binds to antioxidant response elements and regulates the expression of antioxidant genes [56]. On the other hand, it has been suggested that Cd at low concentrations could modulate and inhibit NAD(P)H oxidase activity [57]. It has been reported that this enzyme is the one of the major sources of ROS in the kidney [40,58]. It is known that Ang II increases NAD(P)H oxidase activity through AT1 receptor [41]. We found a decreased NAD(P)H oxidase activity in the group treated with Cd + LOS. In our model, we did not find an increase in oxidative stress, probably due to an antioxidant response of cell.

The mechanism by which Cd decreases cubilin is still unclear. To explore this mechanism, we used LOS, an AT1 receptor antagonist, because endocytosis is regulated by many factors and amongst them Ang II appears to be one of them [32,59]. Moreover, other studies showed that Cd stimulates RAS [33-35] and the use of inhibitors has shown a protective effect on Cd toxicity [60,61]. Regarding endocytosis, Ang II acts on two receptors, AT1 and AT2, both are expressed in proximal tubular cells, and as well as components of RAS; and it has been observed that, in diabetic models, the use of RAS inhibitors (ACE inhibitors and Ang II type 1 receptor) restored the expression of megalin and in consequence albumin reabsorption [31,62]. *In vitro* models have shown that Ang II modulates albumin endocytosis via AT2-Protein Kinase B (PKB) activation [59]. Interestingly, Ang II through AT2 may regulate cubilin receptor and restore albumin endocytosis [47].

## Conclusion

In conclusion, LOS treatment decreased the microalbuminuria induced by Cd by a mechanism independent of megalin, and probably dependent on cubilin, at least at this duration of exposure. Further experiments are required to determine the mechanism by which Cd regulates cubilin expression.

## Competing interests

The authors declare that they have no competing interests.

## Authors’ contributions

MPSS and OCB, conception of the study; MPSS, EMJ, RRM, data collection; MPSS, RRM, EMJ, data analyses; MPSS wrote the first draft, which was refined by contributions of JPC, EMJ, LAM and OCB. All authors were involved in the interpretation of the data, read and approved the final manuscript.

## Pre-publication history

The pre-publication history for this paper can be accessed here:

http://www.biomedcentral.com/1471-2369/14/211/prepub

## References

[B1] ATSDR U.S. Toxicologycal profile for Cadmium Agency for Toxic Substance and Disease Registry 2012 Atlanta: GA: Department of Health and Human Sevices, Public Health Service, Centers for Disease control

[B2] Rzigalinski BA Strobl JS Cadmium-containing nanoparticles: perspectives on pharmacology and toxicology of quantum dots Toxicol Appl Pharmacol 2009 238 3 280 288 10.1016/j.taap.2009.04.010 19379767 PMC2709954

[B3] Storelli MM Marcotrigiano GO Consumption of bivalve molluscs in Italy: estimated intake of cadmium and lead Food Addit Contam 2001 18 4 303 307 11339264 10.1080/02652030120012

[B4] Satarug S Moore MR Adverse health effects of chronic exposure to low-level cadmium in foodstuffs and cigarette smoke Environ Health Perspect 2004 112 10 1099 1103 10.1289/ehp.6751 15238284 PMC1247384

[B5] Reeves PG Vanderpool RA Cadmium burden of men and women who report regular consumption of confectionery sunflower kernels containing a natural abundance of cadmium Environ Health Perspect 1997 105 10 1098 1104 10.1289/ehp.971051098 9349833 PMC1470393

[B6] Vahter M Berglund M Nermell B Akesson A Bioavailability of cadmium from shellfish and mixed diet in women Toxicol Appl Pharmacol 1996 136 2 332 341 10.1006/taap.1996.0040 8619241

[B7] Jarup L Akesson A Current status of cadmium as an environmental health problem Toxicol Appl Pharmacol 2009 238 3 201 208 10.1016/j.taap.2009.04.020 19409405

[B8] Dorian C Gattone VH 2nd Klaasen CD Renal cadmium deposition and injury as a result of accumulation of cadmium-metallothionein (CdMT) by the proximal convoluted tubules–A light microscopic autoradiography study with 109CdMT Toxicol Appl Pharmacol 1992 114 2 173 181 10.1016/0041-008X(92)90066-2 1609408

[B9] Barbier O Jacquillet G Tauc M Cougnon M Poujeol P Effect of heavy metals on, and handling by, the kidney Nephron Physiol 2005 99 4 105 110 10.1159/000083981 15722646

[B10] Soodvilai S Nantavishit J Muanprasat C Chatsudthipong V Renal organic cation transporters mediated cadmium-induced nephrotoxicity Toxicol Lett 2011 204 1 38 42 10.1016/j.toxlet.2011.04.005 21513783

[B11] Thevenod F Nephrotoxicity and the proximal tubule. Insights from cadmium Nephron Physiol 2003 93 4 87 93 10.1159/000070241 12759569

[B12] Klaassen CD Liu J Choudhuri S Metallothionein: an intracellular protein to protect against cadmium toxicity Annu Rev Pharmacol Toxicol 1999 39 267 294 10.1146/annurev.pharmtox.39.1.267 10331085

[B13] Jarup L Berglund M Elinder CG Nordberg G Vahter M Health effects of cadmium exposure–a review of the literature and a risk estimate Scand J Work Environ Health 1998 24 Suppl 1 1 51 9569444

[B14] Gena P Calamita G Guggino WB Cadmium impairs albumin reabsorption by down-regulating megalin and ClC5 channels in renal proximal tubule cells Environ Health Perspect 2010 118 11 1551 1556 10.1289/ehp.0901874 20576581 PMC2974692

[B15] Abouhamed M Wolff NA Lee WK Smith CP Thevenod F Knockdown of endosomal/lysosomal divalent metal transporter 1 by RNA interference prevents cadmium-metallothionein-1 cytotoxicity in renal proximal tubule cells Am J Physiol Renal Physiol 2007 293 3 F705 F712 10.1152/ajprenal.00198.2007 17596526

[B16] Choi JS Kim KR Ahn DW Park YS Cadmium inhibits albumin endocytosis in opossum kidney epithelial cells Toxicol Appl Pharmacol 1999 161 2 146 152 10.1006/taap.1999.8797 10581208

[B17] Gorriz JL Martinez-Castelao A Proteinuria: detection and role in native renal disease progression Transplant Rev (Orlando) 2012 26 1 3 13 10.1016/j.trre.2011.10.002 22137726

[B18] Hoyer JR Seiler MW Pathophysiology of Tamm-Horsfall protein Kidney Int 1979 16 3 279 289 10.1038/ki.1979.130 393892

[B19] Thakker RV The role of renal chloride channel mutations in kidney stone disease and nephrocalcinosis Curr Opin Nephrol Hypertens 1998 7 4 385 388 10.1097/00041552-199807000-00006 9690036

[B20] Birn H Christensen EI Renal albumin absorption in physiology and pathology Kidney Int 2006 69 3 440 449 10.1038/sj.ki.5000141 16514429

[B21] Leheste JR Rolinski B Vorum H Hilpert J Nykjaer A Jacobsen C Aucouturier P Moskaug JO Otto A Christensen EI Megalin knockout mice as an animal model of low molecular weight proteinuria Am J Pathol 1999 155 4 1361 1370 10.1016/S0002-9440(10)65238-8 10514418 PMC1867027

[B22] Christensen EI Birn H Megalin and cubilin: synergistic endocytic receptors in renal proximal tubule Am J Physiol Renal Physiol 2001 280 4 F562 F573 11249847 10.1152/ajprenal.2001.280.4.F562

[B23] Marzolo MP Farfan P New insights into the roles of megalin/LRP2 and the regulation of its functional expression Biol Res 2011 44 1 89 105 10.4067/S0716-97602011000100012 21720686

[B24] Bansal A Gierasch LM The NPXY internalization signal of the LDL receptor adopts a reverse-turn conformation Cell 1991 67 6 1195 1201 10.1016/0092-8674(91)90295-A 1760844

[B25] Christensen EI Birn H Storm T Weyer K Nielsen R Endocytic receptors in the renal proximal tubule Physiology (Bethesda) 2012 27 4 223 236 10.1152/physiol.00022.2012 22875453

[B26] Amsellem S Gburek J Hamard G Nielsen R Willnow TE Devuyst O Nexo E Verroust PJ Christensen EI Kozyraki R Cubilin is essential for albumin reabsorption in the renal proximal tubule J Am Soc Nephrol 2010 21 11 1859 1867 10.1681/ASN.2010050492 20798259 PMC3014001

[B27] Navar LG The intrarenal renin-angiotensin system in hypertension Kidney Int 2004 65 4 1522 1532 10.1111/j.1523-1755.2004.00539.x 15086502

[B28] Kobori H Nangaku M Navar LG Nishiyama A The intrarenal renin-angiotensin system: from physiology to the pathobiology of hypertension and kidney disease Pharmacol Rev 2007 59 3 251 287 10.1124/pr.59.3.3 17878513

[B29] Galle J Reduction of proteinuria with angiotensin receptor blockers Nat Clin Pract Cardiovasc Med 2008 5 Suppl 1 S36 S43 18580865 10.1038/ncpcardio0806

[B30] Carter B Hunsicker L Lewis S Orland B Rodby R Emerging trends for prevention and treatment of diabetic nephropathy: blockade of the RAAS and BP control Evid-Based Approach 2004 10 12 17 10.18553/jmcp.2004.10.S5-A.S12 PMC10437733 15369420

[B31] Tojo A Onozato ML Kurihara H Sakai T Goto A Fujita T Angiotensin II blockade restores albumin reabsorption in the proximal tubules of diabetic rats Hypertens Res 2003 26 5 413 419 10.1291/hypres.26.413 12887133

[B32] Hosojima M Sato H Yamamoto K Kaseda R Soma T Kobayashi A Suzuki A Kabasawa H Takeyama A Ikuyama K Regulation of megalin expression in cultured proximal tubule cells by angiotensin II type 1A receptor- and insulin-mediated signaling cross talk Endocrinology 2009 150 2 871 878 18927221 10.1210/en.2008-0886

[B33] Davalli P Carpene E Astancolle S Viviani R Corti A Cadmium induction of renal and hepatic ornithine decarboxylase activity in the rat. Effects of sex hormones and involvement of the renin-angiotensin system Biochem Pharmacol 1992 44 4 721 726 10.1016/0006-2952(92)90408-B 1510718

[B34] Varoni MV Palomba D Macciotta NP Antuofermo E Deiana G Baralla E Anania V Demontis MP Brain renin-angiotensin system modifies the blood pressure response to intracerebroventricular cadmium in rats Drug Chem Toxicol 2010 33 3 302 309 10.3109/01480540903418496 20429803

[B35] Lall SB Peshin SS Gulati K Khattar S Das N Seth SD Involvement of renin-angiotensin system in hypertensive effect of cadmium in rats Indian J Exp Biol 1997 35 4 338 391 9315231

[B36] Edgell K EPA USEPA Method Study 37 - SW-846 Method 3050 Acid Digestion of Sediments, Sludges, and Soils EPA Contract No 68-03-3254 1989 United States: Environmental Protection Agency, Enviromental Monitoring Systems Laboratory 2 5

[B37] Gerard-Monnier D Erdelmeier I Regnard K Moze-Henry N Yadan JC Chaudiere J Reactions of 1-methyl-2-phenylindole with malondialdehyde and 4-hydroxyalkenals. Analytical applications to a colorimetric assay of lipid peroxidation Chem Res Toxicol 1998 11 10 1176 1183 10.1021/tx9701790 9778314

[B38] Molina-Jijon E Tapia E Zazueta C El Hafidi M Zatarain-Barron ZL Hernandez-Pando R Medina-Campos ON Zarco-Marquez G Torres I Pedraza-Chaverri J Curcumin prevents Cr(VI)-induced renal oxidant damage by a mitochondrial pathway Free Radic Biol Med 2011 51 8 1543 1557 10.1016/j.freeradbiomed.2011.07.018 21839166

[B39] Maldonado PD Molina-Jijon E Villeda-Hernandez J Galvan-Arzate S Santamaria A Pedraza-Chaverri J NAD(P)H oxidase contributes to neurotoxicity in an excitotoxic/prooxidant model of Huntington’s disease in rats: protective role of apocynin J Neurosci Res 2010 88 3 620 629 19795371 10.1002/jnr.22240

[B40] Bedard K Krause KH The NOX family of ROS-generating NADPH oxidases: physiology and pathophysiology Physiol Rev 2007 87 1 245 313 10.1152/physrev.00044.2005 17237347

[B41] Modlinger P Chabrashvili T Gill PS Mendonca M Harrison DG Griendling KK Li M Raggio J Wellstein A Chen Y RNA silencing in vivo reveals role of p22phox in rat angiotensin slow pressor response Hypertension 2006 47 2 238 244 10.1161/01.HYP.0000200023.02195.73 16391171

[B42] Wolf G Ritz E Combination therapy with ACE inhibitors and angiotensin II receptor blockers to halt progression of chronic renal disease: pathophysiology and indications Kidney Int 2005 67 3 799 812 10.1111/j.1523-1755.2005.00145.x 15698420

[B43] Lauwerys RR Bernard A Roels HA Buchet JP Viau C Characterization of cadmium proteinuria in man and rat Environ Health Perspect 1984 54 147 152 6376088 10.1289/ehp.8454147 PMC1568159

[B44] Chaumont A Nickmilder M Dumont X Lundh T Skerfving S Bernard A Associations between proteins and heavy metals in urine at low environmental exposures: evidence of reverse causality Toxicol Lett 2012 210 3 345 352 10.1016/j.toxlet.2012.02.005 22353377

[B45] Gekle M Renal tubule albumin transport Annu Rev Physiol 2005 67 573 594 10.1146/annurev.physiol.67.031103.154845 15709971

[B46] Birn H Fyfe JC Jacobsen C Mounier F Verroust PJ Orskov H Willnow TE Moestrup SK Christensen EI Cubilin is an albumin binding protein important for renal tubular albumin reabsorption J Clin Invest 2000 105 10 1353 1361 10.1172/JCI8862 10811843 PMC315466

[B47] Oroszlan M Bieri M Ligeti N Farkas A Meier B Marti HP Mohacsi P Sirolimus and everolimus reduce albumin endocytosis in proximal tubule cells via an angiotensin II-dependent pathway Transpl Immunol 2010 23 3 125 132 10.1016/j.trim.2010.05.003 20470887

[B48] Prozialeck WC Vaidya VS Liu J Waalkes MP Edwards JR Lamar PC Bernard AM Dumont X Bonventre JV Kidney injury molecule-1 is an early biomarker of cadmium nephrotoxicity Kidney Int 2007 72 8 985 993 10.1038/sj.ki.5002467 17687258 PMC2747605

[B49] Herak-Kramberger CM Brown D Sabolic I Cadmium inhibits vacuolar H(+)-ATPase and endocytosis in rat kidney cortex Kidney Int 1998 53 6 1713 1726 10.1046/j.1523-1755.1998.00914.x 9607204

[B50] Ahn DW Chung JM Kim JY Kim KR Park YS Inhibition of renal Na+/H + exchange in cadmium-intoxicated rats Toxicol Appl Pharmacol 2005 204 1 91 98 10.1016/j.taap.2004.08.021 15781297

[B51] Wang L Chen D Cao J Liu Z Protective effect of N-acetylcysteine on experimental chronic cadmium nephrotoxicity in immature female rats Hum Exp Toxicol 2009 28 4 221 229 10.1177/0960327109102365 19734274

[B52] Lee WK Thevenod F Novel roles for ceramides, calpains and caspases in kidney proximal tubule cell apoptosis: lessons from in vitro cadmium toxicity studies Biochem Pharmacol 2008 76 11 1323 1332 10.1016/j.bcp.2008.07.004 18675256

[B53] Zhou YJ Zhang SP Liu CW Cai YQ The protection of selenium on ROS mediated-apoptosis by mitochondria dysfunction in cadmium-induced LLC-PK(1) cells Toxicol In Vitro 2009 23 2 288 294 10.1016/j.tiv.2008.12.009 19135140

[B54] Thevenod F Friedmann JM Cadmium-mediated oxidative stress in kidney proximal tubule cells induces degradation of Na+/K(+)-ATPase through proteasomal and endo-/lysosomal proteolytic pathways FASEB J 1999 13 13 1751 1761 10506578 10.1096/fasebj.13.13.1751

[B55] Thijssen S Cuypers A Maringwa J Smeets K Horemans N Lambrichts I Van Kerkhove E Low cadmium exposure triggers a biphasic oxidative stress response in mice kidneys Toxicology 2007 236 1–2 29 41 17499415 10.1016/j.tox.2007.03.022

[B56] Chen J Shaikh ZA Activation of Nrf2 by cadmium and its role in protection against cadmium-induced apoptosis in rat kidney cells Toxicol Appl Pharmacol 2009 241 1 81 89 10.1016/j.taap.2009.07.038 19682480

[B57] Groppa MD Ianuzzo MP Rosales EP Vázquez SC Benavides MP Cadmium modulates NADPH oxidase activity and expression in sunflower leaves Biol Plantarum 2012 56 1 167 171 10.1007/s10535-012-0036-z

[B58] Kim SM Kim YG Jeong KH Lee SH Lee TW Ihm CG Moon JY Angiotensin II-induced mitochondrial Nox4 is a major endogenous source of oxidative stress in kidney tubular cells PLoS One 2012 7 7 e39739 10.1371/journal.pone.0039739 22808054 PMC3392275

[B59] Caruso-Neves C Kwon SH Guggino WB Albumin endocytosis in proximal tubule cells is modulated by angiotensin II through an AT2 receptor-mediated protein kinase B activation Proc Natl Acad Sci USA 2005 102 48 17513 17518 10.1073/pnas.0507255102 16293694 PMC1297674

[B60] Fouad AA Jresat I Captopril and telmisartan treatments attenuate cadmium-induced testicular toxicity in rats Fundam Clin Pharmacol 2013 27 2 152 160 10.1111/j.1472-8206.2011.00974.x 21819444

[B61] Fouad AA Jresat I Protective effect of telmisartan against cadmium-induced nephrotoxicity in mice Life Sci 2011 89 1–2 29 35 21620867 10.1016/j.lfs.2011.04.019

[B62] Banes AK Shaw S Jenkins J Redd H Amiri F Pollock DM Marrero MB Angiotensin II blockade prevents hyperglycemia-induced activation of JAK and STAT proteins in diabetic rat kidney glomeruli Am J Physiol Renal Physiol 2004 286 4 F653 F659 10.1152/ajprenal.00163.2003 14678947

